# Hereditary Optic Neuropathies: A Systematic Review on the Interplay between Biomaterials and Induced Pluripotent Stem Cells

**DOI:** 10.3390/bioengineering11010052

**Published:** 2024-01-03

**Authors:** Miguel Ladero, Jose Alberto Reche-Sainz, M. Esther Gallardo

**Affiliations:** 1 FQPIMA Group, Materials and Chemical Engineering Department, Chemical Sciences School, Complutense University of Madrid, 28040 Madrid, Spain; 2 Ophthalmology Unit, Hospital Universitario 12 de Octubre, 28041 Madrid, Spain; 3 Translational Research with iPS Cells Group, Research Institute of Hospital 12 de Octubre, imas12, 28041 Madrid, Spain

**Keywords:** iPSCs, differentiation, transplantation, RGCs, optic neuropathies, LHON, DOA, decellularized extracellular matrix, synthetic polymer, hydrogel, biomaterial

## Abstract

Hereditary optic neuropathies (HONs) such as dominant optic atrophy (DOA) and Leber Hereditary Optic Neuropathy (LHON) are mitochondrial diseases characterized by a degenerative loss of retinal ganglion cells (RGCs) and are a cause of blindness worldwide. To date, there are only limited disease-modifying treatments for these disorders. The discovery of induced pluripotent stem cell (iPSC) technology has opened several promising opportunities in the field of HON research and the search for therapeutic approaches. This systematic review is focused on the two most frequent HONs (LHON and DOA) and on the recent studies related to the application of human iPSC technology in combination with biomaterials technology for their potential use in the development of RGC replacement therapies with the final aim of the improvement or even the restoration of the vision of HON patients. To this purpose, the combination of natural and synthetic biomaterials modified with peptides, neurotrophic factors, and other low- to medium-molecular weight compounds, mimicking the ocular extracellular matrices, with human iPSC or iPSC-derived cell retinal progenitors holds enormous potential to be exploited in the near future for the generation of transplantable RGC populations.

## 1. Introduction

It is foreseen that affections to the retina will grow in the near future, with macular degeneration, diabetic retinopathy, and glaucoma as the most prevalent and leading causes of vision loss and blindness worldwide [1]. Moreover, rare optic neuropathies like Leber Hereditary Optic Neuropathy (LHON) and dominant optic atrophy (DOA) are finding increasing awareness in European society with a clear aim to improve patient access to diagnosis, information, and care. They both share a mitochondrial pathogenesis that leads to the selective loss of retinal ganglion cells (RGCs) and axons [2]. Currently, traditional treatments remain incompetent in terms of restoring visual function. Anatomically, the eye holds the natural advantages of easy accession and operation, immune isolation, and optical transparency, which prioritize ocular diseases for new technology trials, such as stem cell-based treatments [3]. In the last few years, stem cell technologies have revolutionized and hold great promise for the treatment of a wide range of blinding diseases. Among them, induced pluripotent stem cells (iPSCs) are one of the most powerful cell types known. They not only have the possibility of dividing indefinitely, but they have not only the possibility to divide indefinitely; these cells can also potentially differentiate into whatever cell type of the three germ layers [4]. This makes them very useful as models to delve into the physiopathological mechanisms of the diseases, to be used as a platform for drug screenings, or to be applied in cell therapies after being differentiated into the target cell type.

Biomaterials are playing an increasing role in the generation, expansion, differentiation, and transplantation of stem cells and the cells, tissues, and organoids obtained from them as they can provide an adequate environment to maintain stemness during cell self-renewal, guide cell fate during differentiation, and/or maintain the full functionality of target cells and tissues during implantation. Moreover, they should provide the highest accessibility and affordability for a widespread clinical translation of cell-based therapies [5]. Though there are still several needs to be fulfilled, biomaterials are key to creating microenvironments that are identical or similar to the extracellular matrix that surrounds cells, tissues, and organoids. In this regard, decellularized extracellular matrices (dECMs) can provide the adequate physicochemical and biological cues for cell survival, expansion, and differentiation, though decellularization protocols still need optimization, present low productivity, and are specifically dependent on the original tissues that provide them [6]. Matrigel has emerged as a commercial matrix of the dECM type as obtained from Engelbreth–Holm–Swarm mouse sarcoma by Corning Inc. (New York, NY, USA) and is now the substrate of reference for stem cell research. However, due to its origin, this basement membrane extracellular matrix is in contact with diverse cell types (epithelial, endothelial, fat, and smooth muscle cells) and presents a diverse composition from batch to batch that also affects its biochemical properties [7]. As several dECMs, Matrigel presents xenogenic contaminants and a very complex composition of structural proteins, glycoproteins, glycosaminoglycans, and proteoglycans, with 2000 major components and more than 15,000 minor components. Moreover, even if there is a booming market for dECMs from diverse origins (murine, bovine, porcine…), with forecasted compound annual growth rate (CAGR) in the 9.8% range for the period 2023–2030 and a value estimated to be USD 88.73 million by that year, there is still ample space for other natural and synthetic alternatives. In fact, there is a lack of xenogenic-free, highly tuneable, and reproducible materials with a perfectly known chemical composition and physical structure that can act as cell and tissue support and guide and as dECM mimics. In the case of RGCs and neurons, efforts have been directed to synthetic polymers and biopolymers (both mono- and copolymers (polyacrylamide—PAM, polyethyleneglycol—PEG, ethylene–vinylacetate copolymer—EVA, polylactic acid—PLA, poly-ε-caprolactone—PCL, polyglycerolsebacate/poly-ε-caprolactone—PGS/PCL, polylactic-co-glycolic acid/polycaprolactone—PLGA/PCL)) [7,8,9,10] and natural polymers like silk, alginate, hyaluronic acid (HA), chitosan, xanthan gum, and extracellular matrix molecules [11,12,13]. Works reporting on successful differentiation of hiPSCs to RGCs on materials other than Matrigel are scarce [8,9,10]. In these cases, synthetic doped biopolymers seem acceptable, although low final cell production is achieved and cell harvesting from them seems difficult. Knowledge is even more scarce when referring to microbial or crustacean-derived polysaccharides, although their hydrogel structure much more closely resembles extracellular matrix (ECM), making them very promising from a physical perspective [13,14,15]. However, they have a limited capacity for the binding of cells, growth factors, or ECM proteins (except HA) without modification. Moreover, a certain batch-to-batch variability exists.

In this systematic review, we have commented on the two most common inherited optic neuropathies (LHON and DOA) and on the possible application of human induced pluripotent stem cell (hiPSC) technology in the replacement of damaged RGCs in these patients. Finally, diverse types of biomaterials, their main characteristics and their interaction with hiPSCs, retinal progenitors, and RGCs are revised, focusing on their potential use in either RGC generation and/or transplantation.

## 2. Methodology

**Figure 1:**
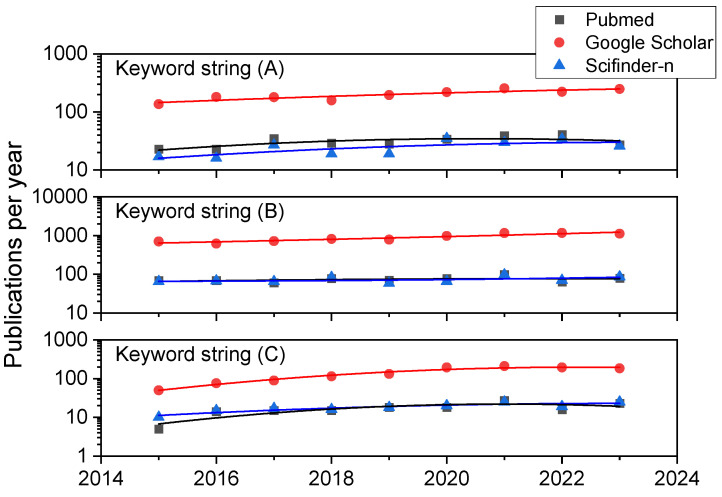
Annual publication trends in the three bibliographic databases consulted. Results for the three general combinations of keywords: (**A**) “Retinal Ganglion Cell AND Hereditary Optic Neuropathy”, (**B**) “Retinal Ganglion Cell AND Stem Cell”, and (**C**) “Retinal Ganglion Cell AND Induced Pluripotent Stem Cell”.

We have carried out a systematic bibliographic analysis in the databases Scifinder-n, Google Scholar, and PubMed databases with the aim of retrieving up-to-date reviews and research articles on HONs (LHON and DOA in particular), iPSC-based therapies, biomaterials, scaffolds and supplementary materials, and chemicals to aid in the creation, expansion, differentiation, and transplantation of retinal ganglion cells or tissues containing them. The time period under surveillance was January 2015–August 2023, except for several key references from 2000 onwards related to HON. The search terms or strings were (A) “Retinal Ganglion Cell AND Hereditary Optic Neuropathy”, (B) “Retinal Ganglion Cell AND Stem Cell”, and (C) “Retinal Ganglion Cell AND Induced Pluripotent Stem Cell”. In combination for string (A), we obtained 280, 1797, and 224 references in PubMed, Google Scholar, and Scifinder-n, respectively. In the first search in Google Scholar and in all cases for search strings (B) and (C) considering manuscripts written in English, results were always in excess of 150 references (as can be appreciated in Figure 1), so we added the following filters as keywords in all the document searches for string (B) and (C): (1) “scaffold” or (2) “hydrogel” or (3)“biomaterial” or (4) “differentiation” or (5) “transplantation”. The combinations (B-1), (B-2), (B-3), (B-4), (B-5), (C-1), (C-2), (C-3), (C-4), and (C-5) resulted in 16, 7, 9, 294, 47, 10, 3, 3, 87, and 47 references using Scifinder-n, while the less detailed, broader search in Google Scholar (where the reference nature cannot be chosen) led to 1400, 1020, 408, 5740, 3400, 376, 218, 122, 1060, and 770 documents. Similar searches in PubMed resulted in 19, 3, 13, 284, 196, 10, 1, 6, 91, and 60 books, documents, clinical trials, meta-analyses, randomized control trials, and narrative and systematic reviews, most of which were research papers and reviews.

A closer look at the publication history of these study results in terms of trends can be appreciated in Figure 1. It compiles data on the yearly number of publications from 2015 to 2023 for keyword strings (A), (B), and (C) using the three literature databases here employed. While a look at former years (those previous to 2015 and since 2000) always shows a dramatic and exponential increment in publications for all the keyword combinations mentioned, even in the last eight years, there is a steady linear growth (in particular for combination C, which includes iPSC as a search term) or a high number of publications is maintained (in particular for combination B, reflecting research on RGCs and stem cells in general), highlighting the rising interest of scientists, physicians, and engineers.

The next step was a detailed title and abstract analysis by the authors of all document groups containing 100 or less documents in order to identify those relevant to HON. This detailed analysis retrieved 89 references cited in this systematic review (between January 2015 and August 2023).

## 3. An Approach to Hereditary Optic Neuropathies

### 3.1. Retinal Ganglion Cells

**Figure 2:**
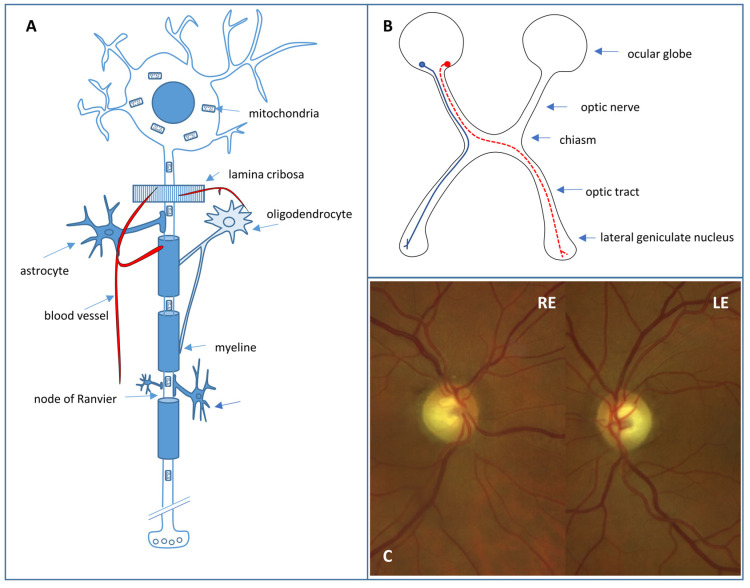
(**A**) Retinal ganglion cell. (**B**) Crossed (red) and uncrossed (blue) fibers in the anterior visual pathway. (**C**) Fundus photograph showing excavated optic nerve heads of a right (RE) and left eye (LE) with temporal pallor in a DOA patient.

Retinal ganglion cell (RGC) loss is the hallmark of optic neuropathies, including glaucoma, where damage to RGC axons occurs at the level of the optic nerve head [16]. RGCs (Figure 2A) are located in the inner retina and act as bridging neurons between the retina and the visual processing centers of the central nervous system (CNS) by receiving electrical signals from the photoreceptors through bipolar cells [17]. There are at least 18 distinct types of RGCs that extract visual stimulus attributes, including spatial contrast, color, motion, fine and coarse details, and light level, before transmitting them to the CNS [17]. These signals are then conveyed via axons, which form the retinal fiber layer and project to the lateral geniculate nucleus through the optic nerve, chiasm, and optic tract, while preserving the retinotopic organization (Figure 2B). In addition, around 1% of RGCs have “non-visual” functions and are intrinsically photosensitive due to the expression of melanopsin. These cells project directly to other nervous structures, such as the suprachiasmatic hypothalamic nucleus (which regulates circadian rhythm) and the pretectal nucleus (which controls the pathway of pupillary reflexes) [17]. Around 1–1.2 million RGC axons exit the ocular globe through 200–300 connective tissue channels that form the scleral cribriform plate. The axons are unmyelinated and surrounded by abundant astrocytes in this laminar portion of the optic nerve. The vascular supply occurs through the arterial circle of Zinn–Haller, which is formed by the short posterior ciliary arteries that are terminal branches. The unique vascularization and peculiar architecture of this portion of the optic nerve play a relevant role in the etiology of some optic neuropathies, such as those of ischemic origin or glaucoma. In the retrolaminar portion of the optic nerve, the nerve fibers are myelinated due to the presence of oligodendrocytes and are also surrounded by meningeal linings.

RGCs have a large number of mitochondria due to their high energy requirement. Therefore, they are especially vulnerable to any alteration in mitochondrial functioning as a result, mainly, of genetic or toxic nutritional disorders. In HON, as an effect of genetic mutations, RGC dysfunction and apoptosis occur, directly affecting bioenergetic production through the impairment of oxidative phosphorylation or acting indirectly through alterations to other processes of mitochondrial homeostasis, such as mitochondrial dynamics (fusion and fission), the maintenance of mitochondrial DNA (mtDNA), interactions with other organelles (endoplasmic reticulum), and the elimination of damaged mitochondria (mitophagy) [18].

### 3.2. The Hereditary Optic Neuropathies

Visual loss caused by optic nerve disorders is a frequent reason for consultation [19]. The clinical manifestations of optic neuropathies are highly heterogeneous, with diverse etiologies including vascular, inflammatory, toxico-nutritional, compressive, infiltrative, or traumatic causes. In the differential diagnosis, hereditary causes which are increasingly important must also be considered. HONs are caused by alterations in nuclear DNA (nDNA) and mtDNA genes that encode proteins essential for mitochondrial functionality. In recent years, great advances in molecular genetics have allowed for the identification of more causative genes, a better understanding of the pathophysiology, as well as the development of more powerful and affordable diagnostic tools. In HONs, RGCs in the prelaminar portion of the papillomacular bundle are particularly vulnerable to mitochondrial homeostasis disturbances, as they are thin, unmyelinated fibers with high energy demands [20,21,22]. Despite the genetic heterogeneity of HONs, central and cecocentral visual field involvement is a common and nearly constant clinical feature. Optic nerve involvement in HON can be a unique and isolated disorder or part of a neurological or systemic syndrome in which optic neuropathy may or may not be a primary phenotypic feature [23] (Table 1). There are two main inherited disorders in which optic neuropathy is the prominent and almost exclusive manifestation. In one of them, the optic neuropathy is insidious and symmetrical (dominant optic atrophy), and in the other, the evolution is subacute and sequential (LHON).

#### 3.2.1. Leber Hereditary Optic Neuropathy

Leber Hereditary Optic Neuropathy (LHON) is one of the most commonly associated mitochondrial diseases with bilateral optic neuropathy [24], with an estimated prevalence of 1 in 25,000 individuals in England and other northern regions of Europe [25]. In total, 90% of cases are due to one of the three point mutations in mtDNA that encode the proteins of complex I of the respiratory chain. These mutations are m.11778G>A (*MT-ND4* gene), m.14484T>C (*MT-ND6* gene), and m.3460G>A (*MT-ND1* gene), which are often found in a homoplasmic state [18,26]. At least 30 other pathogenic mutations have been described. Inheritance is maternal, predominantly affecting males (>90%), with incomplete and variable penetrance due to the influence of nDNA and environmental factors (especially tobacco and alcohol consumption). Only 30% of women with causal genetic mutations will develop the disease, but this percentage rises to 51% in males [18]. Clinically, visual loss usually manifests between the ages of 15–35 years (with a range of 1–87 years) and is typically central, subacute, and painless, with the contralateral eye being affected within a few weeks on average. However, bilateral and simultaneous presentation may occur in 25–50% of cases [18]. Central or centrocecal scotomas are typical in visual field testing, and visual acuity (VA) is usually <20/200. Fundus changes consist of peripapillary telangiectatic microangiopathy with arterial tortuosity and thickening of the fiber layer (pseudopapilledema) in the early stages, which progresses to optic atrophy. Patients with large optic discs, early clinical presentation (before the age of 15), and the m.14484T>C mutation have a better prognosis as they may experience some visual improvement [18,24]. The m.14484T>C mutation has a spontaneous visual recovery rate of 71% compared to the 11% observed for the m.11778G>A mutation [27].

Although LHON is an isolated disorder, some affected patients may have additional very diverse alterations (LHON-plus). These would be produced by mutations that have more systemic repercussions, or in the case of the three main mutations, with the influence of other genes [18]. Systemic manifestations include pre-excitation cardiac syndromes, dystonias, peripheral neuropathies, myopathy, myoclonus, ataxia, epilepsy, psychiatric disorders, dementia, and a multiple sclerosis-like syndrome [18,23,28,29,30].

#### 3.2.2. Autosomal Dominant Optic Atrophy

Autosomal dominant optic atrophy (DOA) is a mitochondriopathy also called type I optic atrophy or Kjer optic neuropathy. The estimated prevalence is 1 per 25,000 inhabitants in Europe and it is even more frequent in some populations, such as Denmark [21,25]. It has an autosomal dominant inheritance with variable clinical penetrance and expressivity, affecting both sexes equally. Visual loss is usually detected at the age of 4–6 years and is initially mild or moderate and bilateral and symmetrical. Sometimes, the individual is not aware of the visual decrease, and the diagnosis is later and even occurs in the context of routine examinations. The evolution is usually slow and insidious (mean VA of 20/100–20/80) [20], producing a progressive thinning of the temporal neuroretinal ring with papillary excavations with ratios greater than 0.5 (Figure 2C). A differential diagnosis must be made mainly with optic neuropathy of compression origin and with low-tension glaucoma [23]. Characteristically, patients present with dyschromatopsia on the blue–yellow axis. Most cases of DOA are due to mutations in the nuclear gene *OPA1* (located on chromosome 3q 28–29) that encodes a GTPase of the inner mitochondrial membrane, which is related to mitochondrial fusion processes [20,22,23,29]. DOA can also be associated with extraocular manifestations (20% of cases) such as sensorineural deafness, cerebellar ataxia, peripheral neuropathy, myopathy, and CPEO (DOA-plus) [23]. Autosomal recessive optic atrophy, or Behr syndrome, is also due to *OPA1* gene mutations transmitted in an autosomal recessive manner or biallelic mutations in which one of them is a hypomorphic variant. Bilateral optic atrophy is much more severe and early and is associated with ataxia, spasticity, and mental retardation [30].

Autosomal dominant optic atrophy and cataract (DOAC) can also be associated with deafness, extrapyramidalism, and ataxia. The inheritance is autosomal dominant and the cataract characteristically appears in the first decade of life. It is due to mutations in the *OPA3* gene (chromosome 19q13.32), which would be involved in the regulation of mitochondrial membrane functions and apoptosis [29,30]. Costeff optic atrophy syndrome is also caused by recessive mutations of the *OPA3* gene and is characterized by early-onset bilateral optic atrophy, somewhat later-onset neurological disorders (ataxia, extrapyramidalism, cognitive deficits), and urinary acid excretion of 3-methylglutaconic acid [30]. Apart from the disorders described, there are many other entities in which optic neuropathy manifests itself in isolation or as part of a systemic syndrome (Table 1).

### 3.3. Treatment of Hereditary Optic Neuropathies

The therapeutic approach must be multidisciplinary and fundamentally supportive. There are still very few evidence-based therapies (mainly idebenone and gene therapy) [19] that can effectively alter the natural history of visual loss in a favorable way. Genetic counseling is of great importance to assess the risk of genetic transmission to offspring.

Idebenone is a short-chain synthetic analogue of coenzyme Q10 (ubiquinone) with hydrophilic qualities such that it more easily crosses the blood–brain barrier. In addition to promoting mitochondrial ATP production by enabling direct electron transfer to complex III of the respiratory chain, it reduces reactive oxygen species [31]. The recommended oral dose is 900 mg daily (300 mg every 8 h). It is the only treatment since 2015 to be approved by the European Medicines Agency (EMA) to be used in LHON, a decision based on the evidence accumulated by the RHODOS clinical trial and other studies [18,19]. The response rate observed was 34% of patients after one year of treatment and 52% after two years, albeit in a highly variable and partial manner. It has been suggested that the probability of visual recovery is greater when treatment is started early, especially before the first year of evolution [32,33]. Although idebenone has provided satisfactory results for treating *OPA1*-associated DOA [34], more evidence is needed in this regard.

#### 3.3.1. Gene Therapy

The RGCs whose axons make up the optic nerve lack regeneration capacity once they undergo apoptosis due to energy deficit in the context of HON [35]. Gene therapies transfer normal genes to cells affected by mutations or deletions, either by viral or non-viral (physical–chemical) means. Specifically, adeno-associated viruses are used to transfer the *MT-ND4* gene affected by the 11,778 mutation type 2 (AAV2) in LHON by intravitreal injection, which has proven to be a highly safe and efficient vector in genetic transduction to RGCs [20,33,35]. Phase III clinical trials, RESCUE (before 6 months of visual loss) and REVERSE (6–12 months after visual loss), revealed bilateral improvement in visual acuity in patients treated with unilateral intravitreal injection at 96 weeks of follow-up, and this improvement was maintained for at least 3 additional years [36,37]. The unexpected bilateral improvement with unilateral injection and the lack of a placebo-controlled arm have prevented this therapy from being approved by the EMA to date [37].

#### 3.3.2. Cell Replacement Therapies: An Alternative Approach

The retina is well adapted for the development of cell therapies for a variety of reasons: (1) most cell types within the retina derive from a common neuronal lineage; (2) the retina is arranged into well-defined layers containing different cell types; and (3) in vivo visualization of the retina up to a cellular resolution is possible throughout any therapeutic period [38]. Because RGC loss can be massive before the diagnosis of visual impairment, cell replacement is one of the most encouraging strategies [39]. However, obtaining pure functional RGC populations followed by proper integration of RGCs within the retina and projection to the brain remain major hurdles in order to translate these techniques into clinical practice [38].

**Table 1:** Genes associated with isolated or syndromic optic nerve atrophy. AD = autosomal dominant. AR = autosomal recessive. DOA = dominant optic atrophy. LHON = Leber Hereditary Optic Neuropathy. XLR = X-linked recessive. XLD = X-linked dominant.

Genes	Locus	Function	Phenotypes	Reference
*OPA1*	3q28–q29	Mitochondrial fusion	DOA (AD)	[40]
			DOA PLUS (AD)	[41]
			Behr syndrome (AR, AD)	[42]
*OPA3*	19q13.2–q13.3	Mitochondrial shape and apoptosis	DOA and cataract (AD)	[43]
			Costeff syndrome (AR)	[44]
*MFN2*	1p36.22	Mitochondrial fusion	Charcot Marie Tooth type 2 A (AD, AR)	[45]
			Hereditary motor and sensory neuropathy type VI (AD)	[46]
*NDUFS2*	2q23.3	Respiratory chain complex I deficiency	LHON fenotype (AR)	[47]
*NDUFS1*	2q33.3.	Respiratory chain complex I deficiency	Optic atrophy and multisystem neurological disorder (AR XLD)	[48]
				[49]
*WFS1*	4q16.1	Endoplasmic reticulum–mitochondria interactions and calcium homeostasis	DOA and hearing loss (AD)	[50]
			Wolfram syndrome (AR)	[51]
*NR2F1*	5q15	Transcriptional regulation	Bosch–Boonstra–Schaaf syndrome (AD)	[52]
*SSBP1*	7q34	mtDNA replication	DOA and retinopathy (AD)	[53]
			DOA, retinopathy, nephropathy, and deafness (AD)	[54]
*SGP7*	16q24.3	Mitochondrial quality control	DOA (AD)	[55]
			Hereditary spastic paraplegia type 7 (AD/AR)	[56]
*AFG3L2*	18q11.21	Mitochondrial quality control	DOA(AR)	[57]
			Spinocerebellar ataxia type 28 (AD)	[58]
			Spastic ataxia type 5 (AR)	[59]
*TIMM8A*	Xq22.1	Translocase of inner mitochondrial membrane	Mohr–Tranebjaerg syndrome (XLR)	[60]
*FXN*	9q21.11	Frataxin (mitochondrial respiratory chain)	Friedrich ataxia (AR)	[61]
*ACO2*	22q13.2	Krebs cycle	Isolated optic atrophy	[62]

## 4. iPSC Technology for Studying and Treating Hereditary Optic Neuropathies

**Figure 3:**
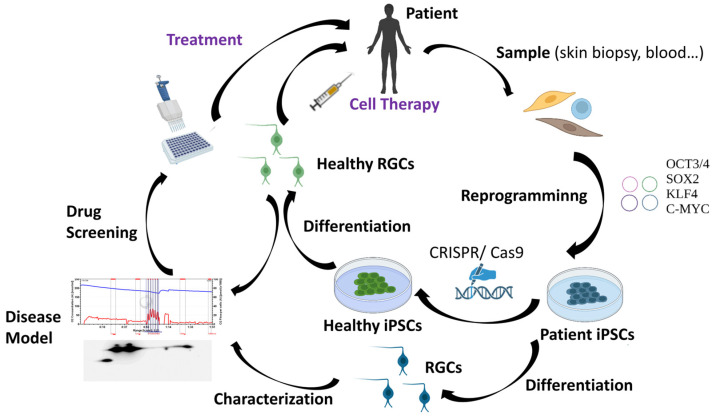
Applications of iPSC technology.

As it has been previously mentioned, RGC loss is the main hallmark of HON. For that reason, many research groups worldwide are working to find methodologies that can allow for the replacement of the damaged RGCs in these patients. However, human RGCs are difficult to access and grow in vitro [63]. Therefore, human pluripotent stem cells, which include human embryonic stem cells (hESCs) and human induced pluripotent stem cells (hiPSCs), represent one of the most promising sources of human RGCs. The discovery of iPSCs has marked a milestone in biomedical research. In 2006, adult murine fibroblasts were successfully reprogrammed into iPSCs by introducing the Yamanaka transcription factors (Oct3/4, Sox2, c-Myc, and Klf4) using retroviral vectors [64]. One year later, hiPSCs were reprogrammed from human adult fibroblasts also using retroviruses [65]. Although iPSCs generated using integrative methodologies can be used to perform basic studies, discover new drugs, and model diseases in vitro, non-integrative methods offer the significant advantage of potentially producing “safe” iPSCs and are thus considered to be more suitable for cell-based therapies [66]. For that reason, the use of non-integrative methods such as Sendai virus, among others, have increased greatly in the last years [67,68,69,70,71]. iPSCs have a morphology and growth behavior typical of ESCs and are also positively stained for typical markers of these cells. iPSCs offer major advantages, including less ethical problems, self-renewability, and pluripotency, which provides them the capacity to potentially differentiate into patient-specific cell types such as RGCs [72]. Cells derived from iPSCs can also be used in regenerative medicine through a variety of approaches including autologous therapies and novel methodologies to edit genomic DNA at a precise locus in iPSCs (e.g., the CRISPR/Cas9 system) [72]. The availability and rapid evolution of this technology has made iPSCs a very promising tool for the generation of patient-specific cellular models and so-called personalized medicine [4]. Patient-derived iPSCs would much better reproduce the specific characteristics of the disease by additionally taking into account the patient’s own genetic background. In this way, iPSCs have enormous utility in helping to understand the interplay between genotype, phenotype, and the response to drugs or potential treatments [4]. Furthermore, iPSCs derived from healthy donors or iPSCs edited with the CRISPR/Cas9 tool could also be an excellent option for cell therapy applications [4,73] (Figure 3).

Regarding HONs, the possibility of obtaining RGCs by differentiation of hiPSCs reprogrammed in vitro from the somatic cells of patients with this disease constitutes a very promising tool for developing models of these types of disorders. Thus, hiPSC-derived RGCs could be used to advance our understanding of the pathophysiological mechanisms of DOA, both as a platform for patient-specific drug testing and as a font to implement the long-desired cell-based therapies. Up to now, several hiPSC lines from patients with optic neuropathies have been created, including LHON and DOA [74,75,76,77,78,79]. However, robust differentiation protocols and accurate implantation techniques are all crucial for the development of a successful cell-based therapy for patients. Many studies have focused on the exploration of technology for the induced differentiation of iPSCs into RGCs, and a series of breakthroughs have been made in the past decade [80]. In fact, several differentiation protocols with diverse strategies have already been developed to generate RGCs from iPSCs in 2D systems [81]. Human RGCs can also be created in human stem cell-derived retinal organoids; however, during long-term organoid culture, RGCs are predominantly lost, limiting studies of RGC maturation and functionality in vitro [82]. In spite of this, iPSC technology holds great promise, though a series of obstacles need to be overcome before any clinical application, such as the refractory nature of the central nervous system to axonal regeneration that could impede the reconnection of new RGC axons to their visual targets [83]. Moreover, purification and enrichment of hiPSC-derived RGCs is essential to ensure the proper functioning of cells and the avoidance of tumor formation after transplantation due to the presence of undesired cell types such as undifferentiated hiPSCs. Currently, the main RGC purification methods include immumopanning, fluorescence-activated cell sorting (FACS), and magnetically activated cell sorting (MACS). Nevertheless, it is also of great importance to explore and find better gene markers for the specific isolation of RGCs [80]. Finally, it is necessary to determine which developmental stage of RGCs is more suitable for in vivo transplantation and which is the best route of administration of these cells to achieve the final aim, the replacement of the lost RGCs. As a proof of concept, mature hiPSC-derived RGCs have been transplanted intravitreally in wild-type C57BL/6J mice. The transplanted RGCs integrated within the ganglion cell layer of host retinas and survived for at least 5 months post transplantation. Moreover, the transplanted RGCs were functional and showed electrophysiologic profiles similar to native mouse RGCs [84]. In conclusion, in spite of the above-mentioned challenges, there is no doubt that hiPSC-based therapy has become the most promising patient-tailored therapeutic approach for the regeneration of RGCs and the improvement of visual function in optic neuropathies.

## 5. The Role of Biomaterials in Optic Neuropathies: Present Developments

**Figure 4:**
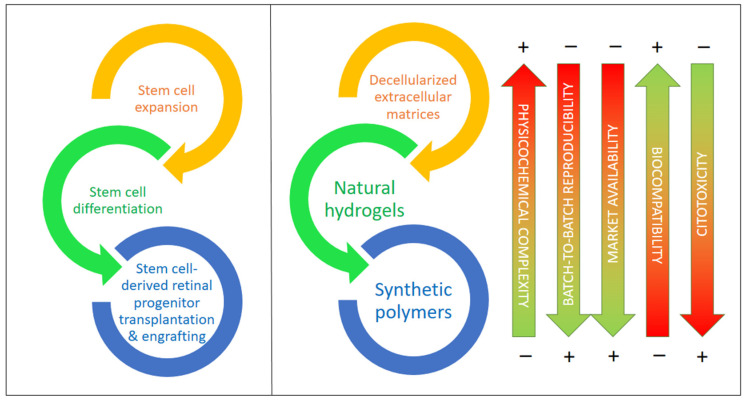
Biomaterials for stem cell expansion, differentiation, and transplantation and the in vivo engrafting of stem cells including types and their most relevant features. Biomaterials are ordered from higher to lower physicochemical complexity and higher to lower biocompatibility.

According to recent studies, supporting materials and carriers would help in the procurement, transplantation, and adaptation of differentiated cells created from iPSCs and other stem cells by providing physical and chemical cues that guide the differentiation of these and other stem cells and stabilize the target cell types, for example, against oxidations due to reactive oxygen species (ROS), which result in apoptosis in most cases [81]. In the last few years, there has been increasing evidence reported on the importance of decellularized extracellular matrix (dEMC) biomaterials, synthetic polymers, and natural hydrogels based on polysaccharides and/or proteins, either alone or in combination with very active small molecules. These biomaterials are key to the protection of stem cells (iPSCs in particular), helping in their expansion, differentiation to RGCs, and, ultimately, in their successful transplantation into the retinal space [85,86]. Figure 4 compiles the main types of biomaterials employed for the expansion, differentiation, transplantation, and engrafting of stem cells, together with a visual assessment of their main advantages and drawbacks.

### 5.1. Extracellular Matrix-Based Biomaterials

A first, and natural, approach to bioscaffolds is the use of the target tissue without cells, that is, the decellularized extracellular matrix (dEMC), as, having relatively low immunogenicity, these materials present the appropriate tridimensional architecture, an adequate chemical composition rich in diverse collagens, elastin, laminin, fibronectin, and other key proteins, as well as a multitude of low-molecular weight peptides and other chemical compounds. Such microenvironments present, therefore, the adequate structural and chemical nature to support stem cells, including iPSCs, and promote their differentiation into functional cells and tissues, helping in the restoration of injured organs, the regeneration of inner tissues, and the replacement of lost tissues and organs [87]. Though the biomimetic identity of dEMC scaffolds is undeniable, serving as a model for any biomaterial, some issues still remain that should be overcome: (1) methods for cell removal still need to be optimized to remove any immunogenic compound and avoid the addition of toxic chemicals; (2) dEMC bioscaffolds should degrade within the proper time, having the appropriate shape and volume for the application; (3) organs and tissues for dEMC construction should be selected to minimize health issues, following a strict quality control process; (4) the inner architecture should be preserved, helping in the innervation and vascularization of tissues and organs for a full functionality; (5) populations of dEMC scaffolds with novel cells and structures still present complexities that need further research; (6) more biological cues of a physical and chemical nature are required to help in the latter issue; and (7) further operational time is required for cellular adhesion, proliferation, and final differentiation and/or the biomaterial transplantation needs of standardization to help in the comparison of in vitro and in vivo results from different research groups to ultimately establish more appropriate conditions for tissue and/or organ regeneration [87,88].

Depending on the dECM origin (organ/tissue or cell culture), diverse techniques can be employed to remove cells, either physical, chemical, or enzymatic and alone or in combination. Procedures that are too mild result in incomplete cell removal, while techniques that are too harsh destroy the innate structure of the tissue, removing key structural cues that are needed in cell repopulation and/or leaving behind toxic compounds. Thus, each dECM has its own optimal fabrication procedure. Regarding the optic nerve, there are only a few works focused on obtaining dECMs [88,89]. Mild procedures based on non-ionic surfactants, like Triton X-100, and their mixture with sodium dodecylsulfate (SDS), an anionic surfactant, provide a decellularized fully integral tissue showing an almost complete absence of cytotoxicity, while SDS alone proved to be deleterious to the optic nerve tissue structure [89]. A progressive protocol using these surfactants on murine and porcine eyes was also successful in obtaining dECM structures that were later on repopulated with retinal pigment epithelial cells (RPECs) and/or with ocular progenitor cells (OPCs) obtained from hiPSCs [90]. Their chemical complexity was evidenced by identifying up to 3837 proteins in the murine eye. In the case of RPECs, they successfully differentiated into a cellular multilayered structure containing well-defined regions containing pigmented cells, photoreceptors, Müller glia, astrocytes, and ganglion cells.

In the case of the optic nerve, to guide and protect RGC axons, providing the adequate support for synaptic connections, two types of dECM biosupports have been developed: an injectable ECM hydrogel that can be placed around or inside the ocular globe and a hybrid electrospun PEUU polymer–ECM tubular sheet [88]. Both materials are suitable for traumatic optic neuropathies (TONs) but, evidently, can be employed in the onset of other types of neuropathies, including HONs. Physical and chemical guiding of RGCs after transplantation can be provided by these supports, as they can deliver and/or contain neurotrophins, stem cells, and inmunotherapeutics. In fact, direct RGC injection usually results in an unordered cell growth and in synaptogenesis failure, as axons have to be directed towards the optic nerve. In particular, the presence of tenascins R and C is key to the correct development of RGC axons, their cellular boundary, and their synaptic connections [91]. Tenascins R and C, together with laminin, fibronectin, and chondroitinsulfate proteoglycans, are of great importance in the preservation of optic nerve structures and stiffness. As reviewed recently by Zhang et al. [92], stiffness is of paramount importance in the transmission and transduction of force signals in all ocular cell types. On the other side, collagen mimetic peptides play a role in the protection of RGCs by restoring collagen fibrillar organization in the ECM [93] peptides obtained from the neural retina and the retinal pigment epithelium (RPE) certainly helps in the maturation of photoreceptors, axon connectivity in the synapsis, and response to light when working with organoids derived from stem cells [85]. Recently, and considering RGCs in particular, a roadmap has been set by the RReSTORe Consortium [94], showing that advances in five key areas are needed to restore the visual pathway damaged by diverse optic neuropathies. These five areas are as follows: (1) RGC development and differentiation, (2) transplantation methods and models, (3) RGC survival, maturation, and host interactions, (4) inner retinal wiring, and (5) eye-to-brain connectivity. Significant progress in these areas needs, at least, the joined efforts of professionals of several fields in medicine, biology, chemistry, material science, and medical engineering.

### 5.2. Synthetic Polymers and Copolymers

Although extracellular matrices of diverse origin and nature, though mainly murine, porcine or bovine, are envisaged as the natural type of structures for cell/tissue/organ production and/or transplantation, they have a natural physicochemical complexity that hinders massive usage at a clinical level. Several reasons and challenges have been mentioned before, though a still notable market growth for dECM is expected in the years to come (9.8% CAGR in the 2023–2030 period). Even with this bountiful prospect, it is evident that less complex, easier to produce 3D cell bioscaffolds are wanted; scaffolds that will mimic dECM structures and, partially, chemical complexities at a fraction of the cost are pursued for scientific and technical reasons. On one side, less complex ECM mimics allow us to understand the diverse roles of physical structures and chemical components in cell–ECM interaction and, ultimately, how it determines the fate of stem cells. On the other hand, they are prone to massive production with relatively simple purification (synthetic polymers) or a more complex downstream but a higher similarity to ECMs (polysaccharide and/or protein-based hydrogels) [95]. Synthetic polymers present the advantage of higher control over their physicochemical properties, while their interaction with cells is inexistent and has to be constructed by grafting diverse biopolymers/biomonomers.

In general, synthetic polymers should demonstrate a local and systemic compatibility if they are to be used as transplantation supports. In this regard, PCL shows promise [96]. Electrospray and fiber structures in these polymers have been proven to be very effective in guiding cell proliferation [97]. Moreover, polyester biomaterials with a fiber structure, such as PCL either alone or mixed with poly(glycerol sebacate) (PGS) or with poly-l-lactide (PLL) or PLGA, have been used as supports for retinal progenitor cells (RPCs) to promote their attachment and expansion [10]. All materials were fabricated with the same electrospinning method and conditions, showing that PGS/PCL scaffolds mixed at a 2:1 weight ratio were superior to the other copolymers and the pure PCL, as indicated by the positive expression of RAX (retina and anterior neural fold homeobox) and NESTIN (neuroectodermal stem cell) markers, as well as RPC attachment and the proliferation rate. Focusing on hiPSC differentiation into RGCs, Chen et al. developed a biomimetic polybenzyl glutamate scaffold. They proceeded from hiPSC-derived neural spheres, observing the outgrowth of neurites and a distinct differentiation to neurons via RNA-seq. Moreover, the ontological and gene network analyses showed that the expressed genes were linked to differentiation into RGCs. Still, a complete RGC characterization was absent [98].

In fact, to facilitate RGC regeneration, a growth stimulation microenvironment should be created. Sluch et al. showed that the combination of PCL nanofiber scaffolds and forskolin, an activator of adenylate cyclase, added in the early stage of iPSC differentiation into RGCs improves this process [99]. Laugther et al. fabricated poly(serinol hexamethylene urea) (PSHU), functionalized it with poly-*N*-isopropylacrylamide (PNIPAAm), and subsequently grafted an RGD motif containing a short peptide via carbodiimide chemistry. After RGD encapsulation into the gel, evident axon growth was perceived by confocal microscopy [100]. Electrospinning has been shown to be a promising fabrication technology for hiPSC expansion and differentiation, but it can be combined with 3D printing to provide axon guidance together with precise positioning of RGCs in the scaffold. This allows for better cell viability and electrophysiology while guiding neurite pathfinding, a key aspect in the eye–brain connection [101]. This creation of long, well-aligned, and organized axons in RGCs has also been observed in an EVA scaffold with parallel grooves created by thermal nano-imprinted lithography [8]. These RGCs demonstrated higher functionality than a control without any topographical cue.

In addition, the presence of chemical and electronic cues also guides axon growth. Chemical neurotrophic factors and guidance cues are commonly used in solution as components of cell culture media. In fact, to increase their stability and create a rich microenvironment, they can be immobilized. Kador et al. immobilized netrin-1 onto electrospun PLA, observing that a netrin-1 gradient along the fibers helped the seeded RGCs to acquire a polarized form, which ultimately led to more mature RGCs, as shown by the developed dendrite form and the electrophysiological function [102]. Electrical stimulation of RGCs on aligned polypyrrole/graphene nanofibers also resulted in better viability with a higher resistance to apoptosis and necrosis, and axon length doubled from 50 to 107 μm [103]. Combining organic photovoltaic materials with adequate electron donors, a photocurrent can be created by laser illumination, simulating an endogenous electric field [104]. This current, again, can increase the growth and maturation of RGCs derived from hiPSCs, stimulating the differentiation of RPCs into RGCs.

### 5.3. Natural Hydrogels Based on Polysaccharides and/or Proteins

Synthetic hydrogels present the advantage of their well-known and tailor-made chemical composition, as well as controllable physical behavior and structure. However, they lack a natural affinity for cells and there are problems that arise due to their low biodegradability/integrability when this factor is critical (for example, for tissue engineering). Their combination with natural hydrogel ingredients (proteins, polysaccharides) or the use of hydrogels based on these polymers can overcome these barriers [105,106]. These natural polymers are biocompatible, biodegradable, and abundant and, if properly chosen and/or modified, they present low immunogenicity. Proteins are very adequate for diverse crosslinking approaches, being also critical for hydrogel–cell interactions. Depending on the selected crosslinking chemistry, their hydrogels or hydrogels containing them and/or synthetic polymers and polysaccharides will present mechanical, structural, and chemical features much resembling those of ECMs [105]. On their side, polysaccharides can present low immunogenicity and proper physical features when crosslinked via physical interactions or through covalent bonds [106]. Immunogenicity is linked to foreign-body response (FBR): once the hydrogel is transplanted, a number of proteins are adsorbed, mediating the interactions with diverse cells of the body. Regulating FBR is critical for cell transplant survival and aims at low protein adsorption, but polysaccharide and protein purity (absence of cytotoxic compounds) is still a costly and difficult-to-achieve aim. As for the cellular cargo within these hydrogels, their ultimate fate is also controlled by the hydrogel 3D macrostructure (stiffness/viscoelasticity) and the presence of cell adhesion motifs, such as peptides with an RGD sequence [106].

To obtain RGCs from stem cells, 2D and 3D methodologies are being developed. Hunt et al. employed alginate-based hydrogels activated at several concentrations with peptides with an RGD motif (GRGDSP-) to encapsulate hiPSC- and hESC-derived embryoid bodies. They also studied hydrogels of hyaluronic acid and hyaluronic acid/gelatin. Results of qRT-PCR indicated that, in the case of the alginate hydrogel with 0.5% *w*/*v* RGD peptides, apart from other markers related to RPCs showing the retinal differentiation of pigmented RPCs and OVs (optic vesicles), the higher expression of the marker *MATH5*, related to RGCs, is evident compared to the control differentiation in liquid media [107]. Apart from hESCs and hiPSCs, dental pulp stem cells (DPSCs) can be differentiated to neuronal lineages and RGCs. In this sense, Roozafzoon et al. studied the creation of RGCs out of DPSCs using both a 2D classical method and a 3D strategy by encapsulating DPSCs from rats in a fibrin hydrogel. In this latter condition, the expression of the markers Pax6, Atoh7, and Brn3b was dramatically higher (2.307-fold, 1.624-fold, and 3.14-fold, respectively) in 3D fibrin hydrogels [108]. The authors concluded that the hydrogels provide a non-toxic 3D microenvironment with structural and mechanical properties similar to those of ECMs, which promotes differentiation to RGCs. A usual combination is alginate and gelatin for the fabrication of hydrogels. Haghighat et al. proved that its combination with retinol promoted the expression of *Nestin*, *RPE65*, and *Rhodopsin* genes and, therefore, the differentiation of mouse MSCs (mesenchymal stem cells) to RGCs [109].

In RPC or RGC transplantation, cell viability and phenotype maintenance are the first objectives, while the second target is cell grafting into the target tissue. It is relatively usual in clinical and research practice to inject cells suspended in buffered liquid media (e.g., PBS) in the vitreal [110] or in the subretinal regions [111]. However, the shear stress that these cells suffer reduces their viability, as shown by Dromel et al. [112]. These authors compared the viability of hRPCs when injected with a 31-gauge needle after being suspended in PBS and after their encapsulation in a hydroxyphenyl propionic acid–gelatin gel (Gtn-HPA) that was enzymatically crosslinked in vivo, on site, after transplantation. Both the suspended and encapsulated RPCs were injected into the sub-retinal eye space of several rats, with a higher number of living engrafted cells and less immune response observed when delivered using the Gtn-HPA gel. This protection also resulted in higher cell viability and proliferation, lower apoptosis, and phenotype maintenance. Gtn-HPA gels appear to be fine vehicles for RPC transplantation as these gels can be crosslinked in situ through the use of small quantities of horseradish peroxidase (HRP) and hydrogen peroxide, showing similar cell survival rates when compared to 2D in vitro controls [112]. Gtn-HPA and HA-Tyr (hyaluronic acid-tyramine) hydrogels, when mixed, are physically and chemically similar to the vitreous and can be crosslinked by HRP-H_2_O_2_ chemistry in situ after injection, facilitating RGC attachment to the inner limiting membrane (ILM) of the retina. Dromel et al. compared the pure gels and their combinations at diverse rates, creating interpenetrating Gtn-HPA/HA-Tyr networks that helped RGCs to engraft to the ILM and connect to the optic nerve by extending long axons. Still, even though the Evans rats used in this study remained healthy, functional and behavioral tests need to be performed.

In Table 2, there is a compilation of the origins and main advantages and disadvantages of the diverse biomaterial families.

## 6. Concluding Remarks and Future Directions

LHON and DOA, the most frequent HONs, are characterized by a degenerative loss of RGCs and are a cause of blindness worldwide. There is currently no cure for vision loss in these diseases due to the fact that RGCs do not regenerate and are not replaced after injury. The combination of hiPSCs or hiPSC-derived retinal progenitor cells with diverse biomaterials acting as supports for cell expansion and differentiation and/or as scaffolds for cell injection, engraftment, and tissular integration, either alone or in the presence of neurotrophic factors and diverse compounds, holds a great promise for retinal or damaged optic nerve regeneration. In this review, we have compiled, in a systematic way, the most recent information regarding the diverse materials and biomaterials of natural, synthetic, and mixed origin available for combination with iPSCs and derived cells with the aim of RGC creation and transplantation. Though recent reviews relative to materials and stem cells are available for more prevalent optic neuropathies, such as glaucoma, this is not the case for rare HONs, such as LHON or DOA.

However, there is still much work to be performed in improving the current protocols for targeted differentiation of hiPSCs to RGCs in order to obtain populations enriched in RGCs without the presence of non-desired retinal cell types. In this sense, nowadays, most protocols are developed for 2D static systems based on Matrigel coating of polystyrene supports. However, most recent evidence advocates for 3D systems where the cells are surrounded by biomaterials that precisely mimic the physical interactions, topography, and biochemical cues present in ECMs. This ECM mimesis still lacks subtleties regarding chemical microenvironments affecting material–cell signaling. The adequate physical support and mass transport reflected in stiffness, viscoelasticity, and permeability in ECMs is not fully achieved. Moreover, topographic cues allowing for directionality require specific shaping technologies to be fully optimized, and electronic and chemical cues are key for axon development and synapsis maturity.

Furthermore, future research directions also include the directed immobilization of neurotrophic and growth factors, such as forskolin, the creation of chemical gradients to stabilize them, and the combination of natural and synthetic polymers to create artificial biomaterials with mechanical, structural, and biochemical features much resembling those of particular extracellular matrices that direct cell engraftment and differentiation after transplantation, avoiding unwanted side effects. In addition, these biomaterials need to be able to integrate (by in vivo crosslinking in the target tissue) or degrade inside without creating toxic residues once they guide RGCs during their integration.

## Figures and Tables

**Table 2:** Biomaterials and synthetic materials employed in the expansion of stem cells (iPSCs in particular) and their differentiation to retinal ganglion cells (RGCs), as well as materials employed in the transplantation of the latter and their precursor cells.

Material Type	Components	Pros	Cons and Needs
**Decellularized extracellular matrix (dECM)**	collagen, elastin, fibronectin, laminin, tenascins, heparan, growth factors	**Adequate** 3D **structure** Presence of **growth factors** and chemical cues Presence of **cell adhesion** **No** ligands or **ligands** with low presence of **immunogenic** cellular **components** **Experience at clinical level** for some tissues (skin: *AlloDerm^®^*, *Oasis^®^*; tendon and ligaments: *GraftJacket^®^*, heart: *CardioCel*^®^…)	Presence of residual **toxic chemicals** used for decellularization (or other toxins) **Low**–medium **batch-to-batch reproducibility** due to origin variability Finely tuned **organ/tissue selection** to avoid risks of **disease transmission** Adequate **material** **shape** for transport and surgery (application) Full **functional integration** (need for vascularization, innervation) **Non-optimal decellularization** leading to tissue architecture degradation
**Synthetic polymers and mixtures**	poly(ε-caprolactone) poly(glycerol sebacate) polylactic-co-glycolic acid poly-(d,l)-lactide polybenzyl glutamate poly(ethylene-co-vinyl acetate) polypyrrole/graphene poly-3-hexylthiophene poly(lactic-co-glycolic acid) poly(serinol hexamethylene urea)	**Simple chemical nature** (but it can be more complex if additives or natural polymers are mixed with synthetic polymers (netrin-1, laminin, fibronectin, cell adhesion peptides, etc.)) **Tunable physical properties** (viscoelasticity, stiffness, permeability, etc.) **Tunable volume shaping and surface topography** (femtosecond laser, two photon lithography, 3D printing, electrospinning) **Batch-to-batch reproducibility**	**Low biocompatibility** (these materials lack the chemical signals typical in dECMs; thus they are usually decorated with laminin, fibronectin, or adhesion peptides (RGD, IKVAV, YIGSR, GVMGFO) typical in integrins, laminins, collagen…, glycans, growth factors, etc.) **Higher toxicity** due to synthesis additives and monomers
**Natural polymers and hydrogels**	polysaccharides (hyaluronic acid, alginate, heparin, carrageenan, fucoidan, dextran, chitosan, cellulose, pullulan, cyclodextrins, etc.) proteins (collagen, gelatin, albumin, elastin, ketatin, resilin, silk)	**Simple chemical nature** (depending on the nature of the polymer and polymer mixtures) **Higher** inherent **biodegradability, biocompatibility**, and **bio-responsive functions** compared to synthetic polymers **Batch-to-batch reproducibility** (but lower than in synthetic polymers)	**Need for intramolecular interaction modulation** (chemical modifications to reach adequate physical properties and structure) **Low stability at physiological conditions** (for example, due to temperature) **Not enough biocompatibility** (still requires polymer mixing (i.e., polysaccharides with proteins) or copolymerization (i.e., with synthetic monomers such as hydroxyphenyl propionic acid or natural monomers such as tyramine) and cell adhesion motifs are still needed)

## Data Availability

Data sharing not applicable.
